# Prognostic significance of ZNF695 in uterine corpus endometrial carcinoma: single-cell and immune infiltration analyses

**DOI:** 10.3389/fonc.2026.1827186

**Published:** 2026-08-10

**Authors:** Huijuan Jiang, Junling Zhu, Zhang Lei, Tuxunayi Yimamu, Qiannan Li, Zuoerbiya Maimaiti, Ainiwaerjiang Abudourousuli

**Affiliations:** 1 Department of Pathology, The First People`s Hospital of Kashi Prefecture, Kashi, Xinjiang, China; 2 School of Medicine, Kashi University, Kashi, Xinjiang, China; 3 Institute of Etiology and Prevention of Metabolic Diseases in Pamir Plateau Area, Kashi University, Kashi, Xinjiang, China; 4 Department of Gynecology, The First People’s Hospital of Kashi Prefecture, Kashi, Xinjiang, China; 5 Kashi Prefecture Cancer Research Institute, The First People`s Hospital of Kashi Prefecture, Kashi, Xinjiang, China

**Keywords:** biomarker, prognosis, single-cell analysis, tumor immune microenvironment, uterine corpus endometrial carcinoma, ZNF695

## Abstract

**Background:**

Zinc Finger Protein 695 (ZNF695) has been reported as a prognostic indicator in several cancers; however, its clinical implications and functional contributions within uterine corpus endometrial carcinoma (UCEC) have not yet been elucidated. Herein, the prognostic significance of ZNF695 in UCEC and its potential involvement in immune infiltration were examined.

**Methods:**

Bioinformatics analyses were conducted utilizing The Cancer Genome Atlas (TCGA) data to evaluate ZNF695 expression, its impact on survival, and patient clinicopathological features. ZNF695 protein expression and subcellular localization were validated using immunohistochemistry (IHC) on a UCEC tissue microarray. To dissect tumor heterogeneity and immune microenvironment, single-cell RNA-seq data from the Gene Expression Omnibus (GEO) were analyzed. The immune infiltration patterns were evaluated utilizing the CIBERSORT algorithm. Three prognostic nomograms were developed to assess UCEC patient survival at the 1-, 3-, and 5-year marks. Furthermore, *in vitro* cellular assays were conducted to validate ZNF695 expression and its biological roles in UCEC cells via siRNA knockdown.

**Results:**

ZNF695 was significantly elevated in UCEC and associated with various clinicopathological features including age, body weight, menopause status, clinical stage, and histological grade. Functional enrichment analyses indicated that ZNF695 was involved in key biological functions, including endopeptidase regulator activity, estrogen signaling pathway and MYC targets. Additionally, single-cell subgroup analysis isolated different cell populations with high ZNF695 expression, underscoring its role in tumor microenvironment. Immune infiltration analyses indicated negative correlations between ZNF695 and various immunosuppressive immune cell subtypes. Elevated ZNF695 expression was strongly correlated with unfavorable clinical outcomes in UCEC. The nomograms for overall survival (OS), disease specific survival (DSS), and progression free interval (PFI) demonstrated promising predictive efficacy, with concordance indices (C-indexes) of 0.631, 0.719, and 0.787, respectively. *In vitro* functional assays demonstrated that siRNA-mediated ZNF695 knockdown markedly impaired the proliferation, migration, and invasion of UCEC cells, accompanied by cell-cycle arrest and altered apoptosis.

**Conclusion:**

ZNF695 serves as an independent prognostic biomarker for UCEC, with its potential as a molecular marker validated experimentally. These findings emphasize the critical role of ZNF695 in regulating the immune landscape of UCEC, warranting studies to elucidate underlying molecular mechanisms and explore its clinical translational implications.

## 1 Introduction

Uterine corpus endometrial carcinoma (UCEC) is among the most frequently identified malignant tumors in the female reproductive system, contributing a substantial proportion of gynecologic cancer-related deaths globally (1, 2). The incidence of UCEC exhibits a steady increase over the past decade, a trend closely associated with the global increase in obesity and metabolic syndrome (3, 4). Excessive adiposity promotes estrogen-dependent endometrial hyperplasia and carcinogenesis through multiple mechanisms, including insulin resistance, chronic inflammation, and dysregulated adipokine signaling (5–7). Reflecting this growing burden, UCEC was ranked the sixth most commonly diagnosed cancer in women globally in 2022 (8). Similarly, in China, UCEC maintains a notably high incidence, consistently ranking between eighth and tenth among all female malignancies (9). While early-stage UCEC achieves favorable outcomes through surgery and adjuvant therapy, the management of advanced or recurrent disease continues to pose significant clinical challenges (10). Notably, for patients with metastatic UCEC, the 5-year survival rate remains under 40%, highlighting a critical medical need (11). This prognostic disparity underscores the need to discover new prognostic indicators and therapeutic targets, which are essential for refining risk stratification and advancing personalized treatment approaches.

Zinc finger proteins (ZFPs), a substantial family of transcription factors, play critical roles in cancer initiation and progression by regulating processes, including cell cycle, epithelial-mesenchymal transition, and immune system responses (12, 13). Within this family, ZNF695 has recently emerged as a potential oncogene. Initially characterized as a transcriptional repressor, ZNF695 features a conserved C2H2-type zinc finger domain and is predicted to exert diverse regulatory effects on gene transcription and related cellular processes (14). Prior studies have validated the prognostic relevance of ZNF695 in esophageal cancer, cervical squamous cell carcinoma, colorectal cancer, and ovarian cancer, where its overexpression correlates with unfavorable survival outcomes (15–17). In these malignancies, elevated ZNF695 expression is associated with advanced tumor stages, increased proliferation, and unfavorable survival outcomes, potentially through the modulation of oncogenic pathways such as PI3K/AKT and interactions with the tumor immune microenvironment (18). Nevertheless, the clinical relevance and biological functions of ZNF695 in UCEC remain entirely unexplored.

To fill this important gap, the current study comprehensively assessed the expression and prognostic significance of ZNF695 in UCEC. Additionally, we conducted single-cell RNA sequencing analysis, functional enrichment analyses (including Gene Ontology (GO), Kyoto Encyclopedia of Genes and Genomes (KEGG), and Gene Set Enrichment Analysis (GSEA), and assessments of immune infiltration to decipher the potential function of ZNF695 in tumorigenesis and microenvironment regulation. We also performed *in vitro* experiments to confirm ZNF695`s role in UCEC cell malignant behaviors. Finally, we validated its prognostic significance using survival analysis. Our findings highlight ZNF695 as a prognostic biomarker in UCEC, providing insights into its functional mechanisms and clinical relevance.

## 2 Materials and methods

### 2.1 Data acquisition and preprocessing

RNA-seq data and relevant clinical information for this study were obtained from TCGA. The initial dataset included 554 primary tumor specimens and 32 adjacent normal tissue samples. We excluded 28 samples that exhibited non-endometrioid histology characteristics and/or lacked complete survival information, resulting in a final cohort of 504 UCEC cases for subsequent analysis. Additionally, gene expression datasets GSE183185 and GSE106191 were obtained from the Gene Expression Omnibus (GEO) database (https://www.ncbi.nlm.nih.gov/geo/) and used for differential expression and receiver operating characteristic (ROC) analysis.

### 2.2 Tissue microarray and immunohistochemistry

The UCEC tissue microarray was obtained from Shanghai Zhuoli Biological Co., Ltd. (catalog number: ZL-UteS961). This array consisted of 48 UCEC specimens alongside 48 corresponding adjacent non-tumor tissue samples, thereby enabling a comprehensive evaluation of ZNF695 expression across different stages of cancer progression. Ethics approval for this research was obtained from the Ethics Committee of Shanghai Zhuoli Biological Co., Ltd. (Approval Number: LLSM-15-01-250721). Given that this study was retrospective and employed anonymized patient records, the necessity for individual informed consent was exempted. All methodologies adhered strictly to the ethical principles delineated in the Declaration of Helsinki. The detailed procedures of IHC were performed as previously described in our published work (19).

### 2.3 Analysis of single-cell transcriptomic data

Single-cell RNA-seq data from five UCEC samples were acquired from GEO database under accession number GSE173682. Quality control was performed by filtering out doublets using DoubletFinder (v2.0.3) and excluding cells with high mitochondrial gene content (>15%) or outlier numbers of detected genes (<200 or >6000). Ribosomal RNA (rRNA)-related genes were also excluded to reduce technical noise. Batch effects were modified via the Harmony algorithm, and subsequent clustering and visualization were carried out with Seurat (v4.0.1). Cell types were annotated by combining reference-based annotation (SingleR) with classical marker genes. Single-cell virtual knockdown of ZNF695 was performed using scTenifoldKnk (v0.1.0) (20). Briefly, a single-cell gene regulatory network (scGRN) was constructed from the filtered expression matrix; ZNF695 was then computationally silenced by setting its outgoing regulatory edges to zero. Differentially regulated genes were identified by comparing the perturbed network with the wild-type scGRN, enabling inference of associated pathway associations.

### 2.4 Functional enrichment analysis

To clarify the potential biological roles and signaling pathways linked with ZNF695 expression, differential expression gene screening was performed prior to GSEA. When comparing the ZNF695-high expression group with the ZNF695-low expression group, a total of 312 genes showed distinct expression based on the screening criteria of Padj < 0.05 and |Log2-FC| > 2. Subsequently, GSEA was conducted using the Hallmark gene sets from MSigDB (v7.4). This method evaluates the distribution of pre-defined gene sets within a ranked gene list to identify significantly enriched biological processes. Gene sets with a |normalized enrichment score (NES)| > 1.5 and a false discovery rate (FDR) < 0.25 were considered statistically significant. Visualization of the results was performed using the enrichplot R package (v1.18.1) to generate enrichment plots, facilitating the interpretation of key pathways modulated by ZNF695.

### 2.5 Analysis of immune cell infiltration

The immune cell profiles in the tumor microenvironment was assessed using two complementary computational approaches: ssGSEA was used for evaluating innate immune activity and overall immune infiltration, and CIBERSORTx was employed for quantifying the relative proportions of specific lymphocyte subsets. Spearman’s rank correlation analysis was employed to examine the associations between ZNF695 expression and immune cell abundances.

### 2.6 Development and validation of the nomograms

Three prognostic nomograms were developed based on independent risk variables that were determined via multivariate Cox regression analysis. The predictive accuracy of the nomograms were assessed using calibration curves, and the concordance index was calculated to quantify model discrimination. All nomograms and calibration plots were generated using the RMS package in R. To further verify the predictive accuracy of the established models, time-dependent ROC analysis were implemented using the timeROC package.

### 2.7 *In vitro* validation of ZNF695

*In vitro* experiments were performed using two UCEC cell lines (HEC-1-A, Ishikawa) and the normal human endometrial cell line hESC as the control. For loss-of-function assays, ZNF695 expression was knocked down by transfection with commercially synthesized small interfering RNA (siRNA) targeting ZNF695. The siRNA sequences and qPCR primers are listed in **Supplementary Table 1** and **Supplementary Table 2**, respectively.

#### 2.7.1 Cell proliferation assay

The proliferative capacity of UCEC cells was evaluated by Cell Counting Kit-8 (CCK-8) assay. Cells were seeded at a density of 5×10^5^ cells per well and incubated under standard culture conditions.

#### 2.7.2 Wound healing assay

Cell migration ability was assessed by wound healing assay. Cells were plated in 6-well plates at a density of 5×10^5^ cells per well and cultured to 80–90% confluence. A scratch was created using a sterile pipette tip, and images were acquired at 0 h and 24 h.

#### 2.7.3 Transwell invasion assay

Cell invasion was determined using Transwell chambers (8.0 μm) pre-coated with Matrigel. Cells were seeded into the upper chamber at a density of 2×10^5^ cells per chamber. After incubation, invaded cells were fixed, stained, and counted under microscopy.

#### 2.7.4 Flow cytometric analysis of cell cycle and apoptosis

Cell cycle distribution and apoptosis were analyzed by flow cytometry. For each sample, 5×10^5^ cells were collected, stained, and processed according to the manufacturer`s instructions. All flow cytometric data were analyzed using BD CellQuest Pro software (BD Biosciences, San Jose, CA, USA).

### 2.8 Statistical analysis

Statistical analyses were carried out with R software (ver. 3.6.3). To assess the associations between ZNF695 expression and clinicopathological characteristics, the Wilcoxon rank-sum test, Chi-square test, or Fisher’s exact test was used as appropriate. Furthermore, logistic regression analysis was performed to assess the independence of these associations. Survival probabilities were examined through Kaplan-Meier method, and comparisons were made via the log-rank test. Both univariate and multivariate Cox proportional hazards models were employed to select variables that were independently correlated with OS, DSS, and PFI.

## 3 Results

### 3.1 ZNF695 is significantly overexpressed in UCEC

**Figure 1:**
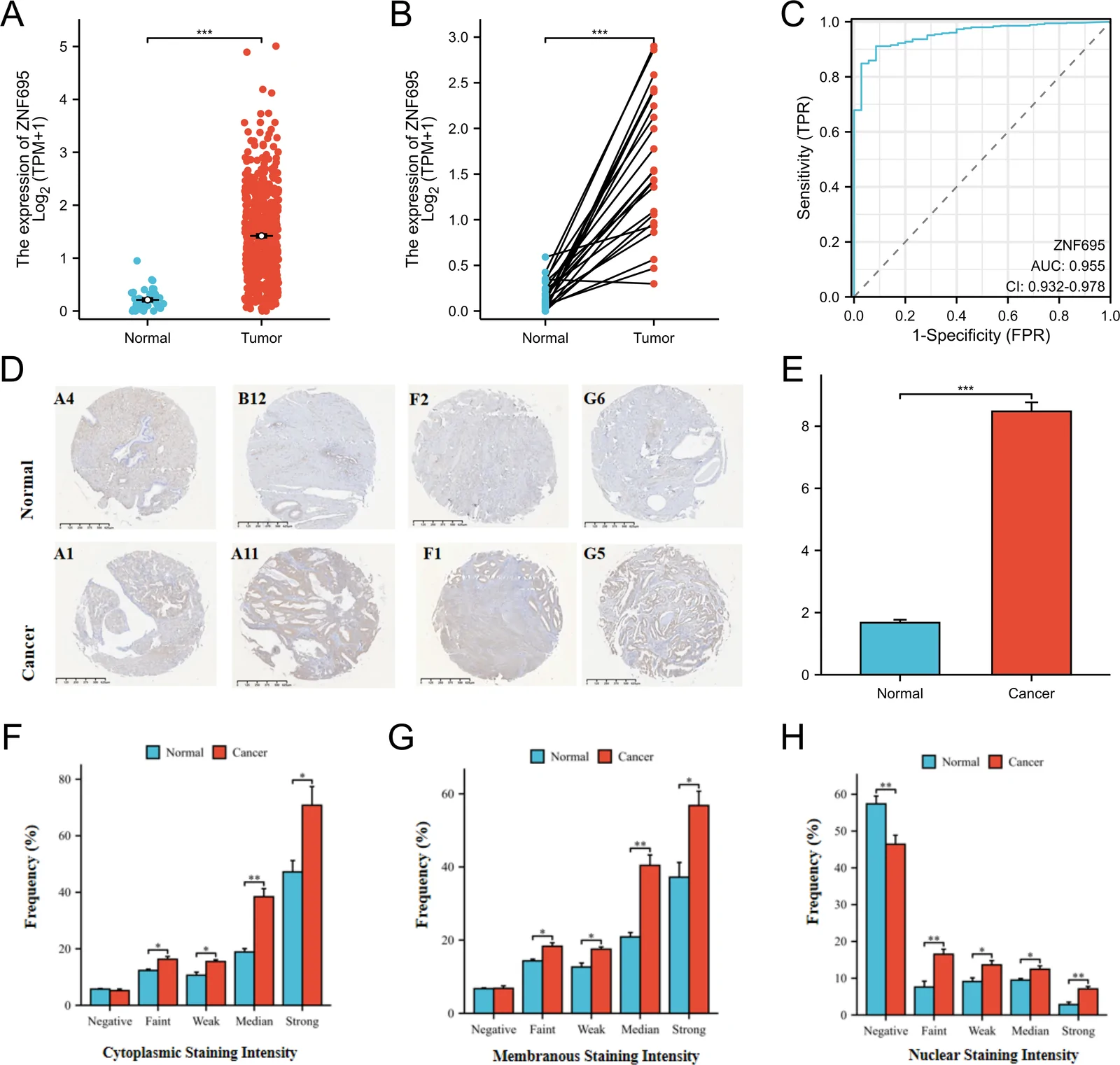
High ZNF695 expression in UCEC. **(A)** ZNF695 expression in the TCGA-UCEC cohort. **(B)** ZNF695 expression in matched samples from TCGA-UCEC. **(C)** ROC curve of ZNF695 in UCEC. **(D)** Representative IHC image of ZNF695 in UCEC tissue microarray. **(E)** Statistical validation of ZNF695 expression in UCEC. **(F)** Cytoplasmic staining intensity. **(G)** Membrane staining intensity. **(H)** Nuclear staining intensity. * p<0.05, ** p<0.01, *** p<0.001.

An initial pan-cancer analysis of ZNF695 expression was conducted using the TIMER2.0 database (**Supplementary Figure 1A**). We further performed pan-cancer analyses using TCGA data. Both pan-cancer analyses revealed that ZNF695 mRNA was markedly elevated across various malignancies, including UCEC (**Supplementary Figure 1B**). ZNF695 expression in 16 paired cancer types also indicated similar results (**Supplementary Figure 1C**). Our analysis of the TCGA-UCEC dataset indicated that ZNF695 was upregulated in UCEC cases (**Figure 1A**); this differential expression was also observed in matched tumor-normal pairs (p <0.001; **Figure 1B**). ROC analysis showed that ZNF695 has diagnostic potential for UCEC (AUC = 0.955, 95% CI: 0.932 - 0.978; **Figure 1C**). Additionally, our analysis in GSE183185 and GSE106191 datasets confirmed elevated expression of ZNF695 in UCEC, with AUC values of 0.861 and 0.628, respectively (**Supplementary Figures 2A–D**). Our tissue microarray analysis indicated elevated expression of ZNF695 in UCEC samples (**Supplementary Figures 3A–E**; **Supplementary Table 3**). IHC validation revealed prominent cytoplasmic staining of ZNF695 in tumor tissues, whereas adjacent non-tumor tissues showed minimal expression (**Figures 1D, E**). Within UCEC tissues, ZNF695 predominantly exhibited diffuse cytoplasmic localization, weak expression on the cell membrane, and negligible detection in the nuclear compartment (**Figures 1F–H**). Taken together, these results consistently demonstrate that ZNF695 is significantly overexpressed in UCEC, which may play a role in the pathogenesis of this malignancy.

### 3.2 ZNF695 expression correlates with multiple clinical features in UCEC

**Figure 2:**
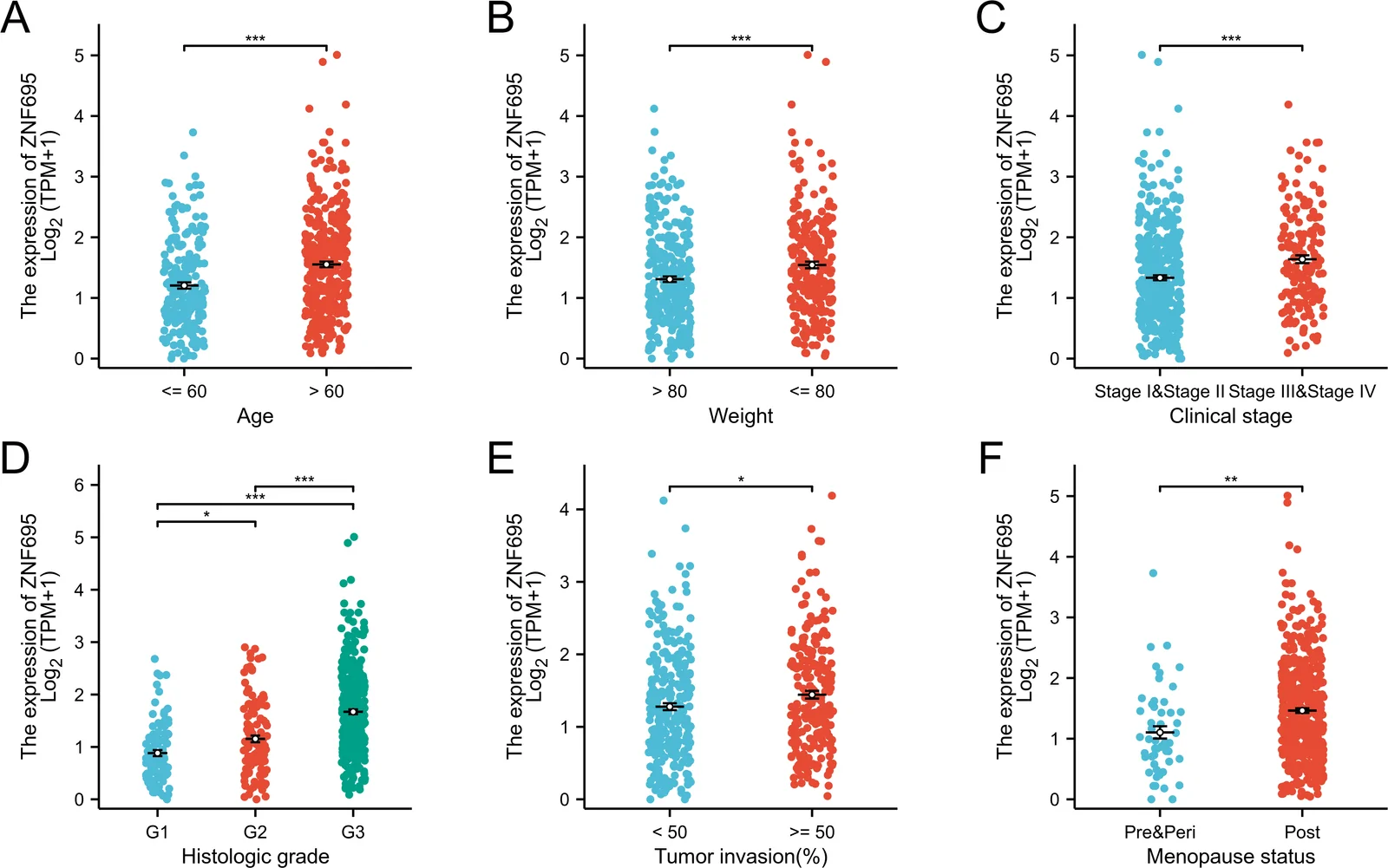
The associations between ZNF695 expression and several UCEC clinicopathological characteristics. **(A)** Age. **(B)** Body weight. **(C)** Clinical stage. **(D)** Histological grade. **(E)** Tumor invasion. **(F)** Menopausal status. * p<0.05, ** p<0.01, *** p<0.001.

We investigated the association between ZNF695 expression levels and various clinical features of UCEC patients (**Table 1**). Our analysis revealed strong correlations between ZNF695 expression and advanced age, weight, clinical stage, histological grade, tumor invasion depth and menopause status (**Figures 2A–F**). Subsequent logistic regression analysis further validated these associations (**Table 2**). Additional analyses of TCGA-UCEC data showed that ZNF695 was positively associated with CDKN2A and MKI67, and negatively associated with PGR (**Supplementary Figures 3F–H**). These results indicate that ZNF695 expression is closely tied to multiple clinicopathological features of UCEC, suggesting that it could act as a valuable marker to predict disease progression.

**Table 1:** Association of ZNF695 expression with UCEC clinical features.

Characteristics	ZNF695-low (n=277)	ZNF695-high (n=277)	p
Age (years)			< 0.001
≤ 60	126 (22.9%)	81 (14.7%)	
> 60	149 (27%)	195 (35.4%)	
Body weight (kg)			0.011
≤ 80	107 (20.2%)	136 (25.7%)	
> 80	158 (29.8%)	129 (24.3%)	
Clinical stage			0.001
I	193 (34.8%)	150 (27.1%)	
II	24 (4.3%)	28 (5.1%)	
III	48 (8.7%)	82 (14.8%)	
IV	12 (2.2%)	17 (3.1%)	
Height (cm)			0.817
≤ 160	121 (23%)	126 (24%)	
> 160	139 (26.5%)	139 (26.5%)	
BMI			0.117
≤ 30	97 (18.6%)	115 (22.1%)	
> 30	163 (31.3%)	146 (28%)	
Histological type			< 0.001
Endometrial	243 (43.9%)	169 (30.5%)	
Serous/mixed	34 (6.1%)	108 (19.5%)	
Residual tumor			0.198
R0	200 (48.2%)	177 (42.7%)	
R1/R2	16 (3.9%)	22 (5.3%)	
Histologic grade			< 0.001
G1	81 (14.9%)	18 (3.3%)	
G2/G3	192 (35.4%)	252 (46.4%)	
Tumor invasion depth (%)			0.111
< 50	149 (31.3%)	112 (23.5%)	
≥ 50	107 (22.5%)	108 (22.7%)	
Menopause status			0.008
Pre/peri	35 (6.9%)	17 (3.4%)	
Post	218 (43%)	237 (46.7%)	
Diabetes			0.931
No	166 (36.6%)	163 (36%)	
Yes	62 (13.7%)	62 (13.7%)	
Hormone therapy			0.776
No	153 (44.2%)	146 (42.2%)	
Yes	23 (6.6%)	24 (6.9%)	

Bold values indicate statistically significant difference (p<0.05).

**Table 2:** Investigating ZNF695 in UCEC via Logistic regression analysis.

Clinical features	Total	OR (95% CI)	p
Age (> 60 vs. ≤ 60)	551	2.036 (1.433 – 2.893)	**< 0.001**
BMI (> 30 vs. ≤ 30)	521	0.756 (0.532 – 1.073)	0.117
Height (cm) (> 160 vs. ≤ 160)	525	0.960 (0.682 – 1.353)	0.817
Weight (kg) (> 80 vs. ≤ 80)	530	0.642 (0.455 – 0.906)	**0.012**
Clinical stage (III/IV vs. I/II)	554	2.012 (1.380 – 2.933)	**< 0.001**
Histologic type (Serous/Mixed vs. Endometrial)	554	4.567 (2.964 – 7.039)	**< 0.001**
Histologic grade (G2/G3 vs. G1)	543	5.906 (3.428 – 10.177)	**< 0.001**
Menopause status (Post vs. Peri/Pre)	507	2.238 (1.219 – 4.111)	**0.009**
Diabetes (Yes vs. No)	453	1.018 (0.674 – 1.539)	0.931

Bold values indicate statistically significant difference (p<0.05).

### 3.3 ZNF695 shows cell-type-specific expression in UCEC

**Figure 3:**
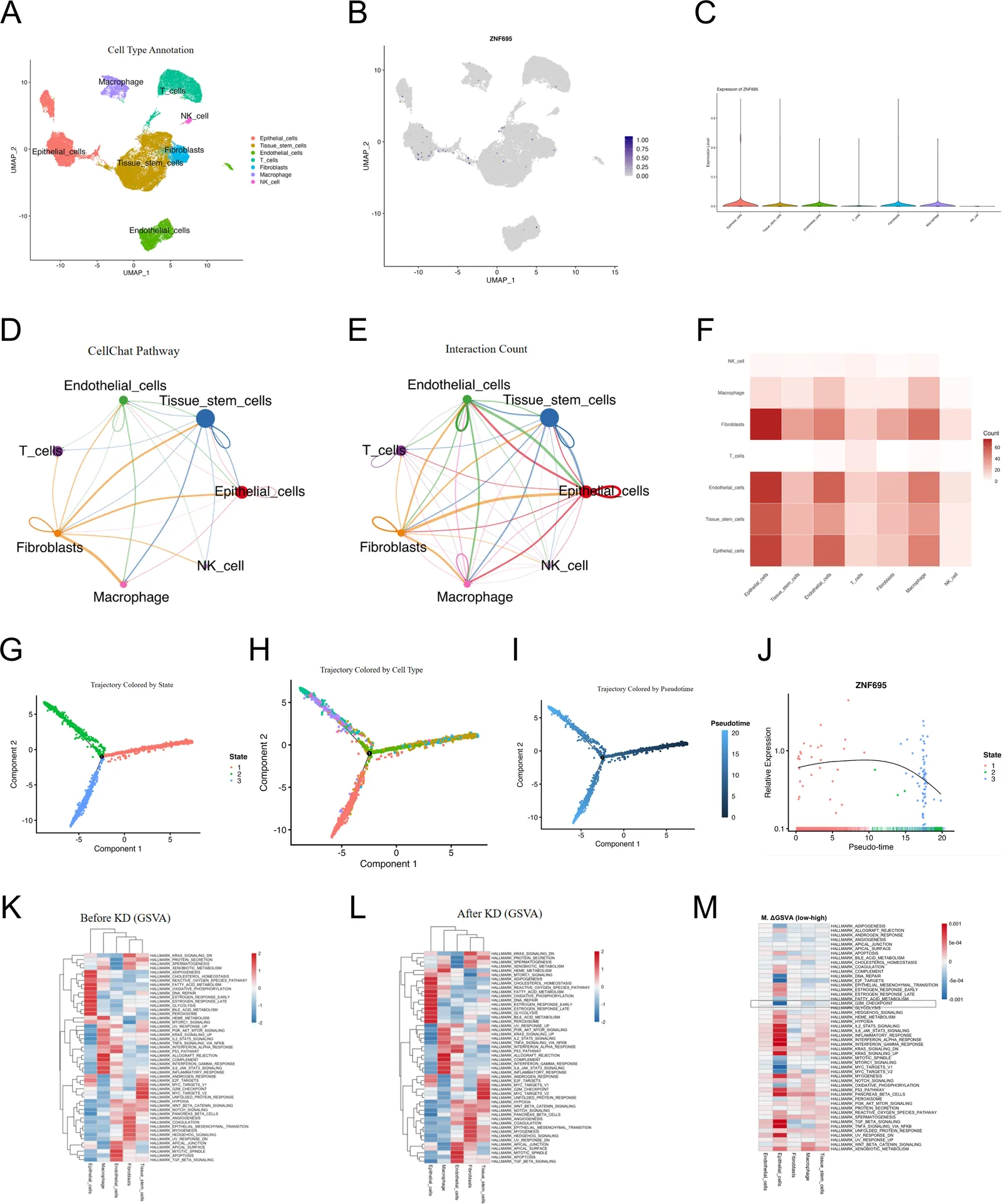
Single-cell analysis of ZNF695 in UCEC. **(A)**UMAP plot visualization of cell subtypes. **(B)** Expression of ZNF695 in various cell clusters. **(C)** Validation of ZNF695. **(D)** Number of interactions. **(E)** Interaction strength. **(F)** Communication pattern of collagen pathway. **(F)** Heatmap showing quantitative counts of cell-cell interactions. **(G)** Pseudotime trajectory colored by cellular states. **(H)** Trajectory annotated by cell subtypes. **(I)** Trajectory gradient-colored by pseudotime values. **(J)** ZNF695 expression dynamics across pseudotime. **(K)** GSVA pathway enrichment before ZNF695 virtual knockdown. **(L)** GSVA pathway enrichment after ZNF695 virtual knockdown. **(M)** Differential GSVA heatmap comparing pre- versus post-KD enrichment scores.

To characterize ZNF695 expression at single-cell resolution, we analyzed the GSE173682 scRNA-seq dataset of UCEC. First, unsupervised clustering identified seven major cell populations: epithelial cells, tissue stem cells, endothelial cells, T cells, fibroblasts, macrophages and NK cells (**Figure 3A**). We then mapped ZNF695 expression across these cell subtypes: UMAP visualization showed distinct ZNF695-high cell clusters (**Figure 3B**), and quantitative analysis confirmed that ZNF695 was predominantly enriched in epithelial cells, tissue stem cells, endothelial cells, fibroblasts and macrophages (**Figure 3C**). Furthermore, CellChat analysis of intercellular communication predicted substantial crosstalk among epithelial cells, endothelial cells, fibroblasts and macrophages (**Figures 3D–F**). To further explore the dynamic role of ZNF695 during tumor progression, we performed pseudotime trajectory analysis on the scRNA-seq data. The trajectory revealed distinct cellular states (**Figure 3G**), with tissue stem cells and epithelial cells occupying key branching points (**Figure 3H**). Notably, ZNF695 expression exhibited a dynamic pattern along the pseudotime axis, peaking at an intermediate stage and declining thereafter, implying a context-dependent function of ZNF695 during cell state transitions (**Figures 3I, J**). To dissect the functional pathways associated with ZNF695 expression, we conducted Gene Set Variation Analysis (GSVA) on the scRNA-seq dataset. GSVA revealed that ZNF695-high cells were significantly enriched in pathways related to epithelial-mesenchymal transition (EMT), cell cycle progression, and inflammatory response (**Figure 3K**). To validate the regulatory role of ZNF695, we performed virtual knockdown (KD) of ZNF695 in the scRNA-seq data and repeated GSVA. Post-KD GSVA showed a marked reduction in the enrichment of EMT and cell cycle pathways, alongside a dampening of inflammatory response signatures (**Figure 3L**). Direct comparison of GSVA profiles before and after ZNF695 KD confirmed the downregulation of oncogenic and inflammatory pathways, while tumor suppressor pathways were modestly upregulated (**Figure 3M**). Notably, pathway enrichment divergence analysis post-virtual KD identified the G2M checkpoint pathway as the most prominently altered cell cycle-related program, prompting us to perform targeted single-cell analysis to characterize ZNF695-mediated regulation of core G2M checkpoint effectors. We focused on two positive regulators of G2M progression—CDK1 and CCNB1—and a key negative regulator, CDKN1A (p21). First, UMAP visualization of these critical molecules revealed cell-type-specific spatial expression patterns across the UCEC single-cell landscape (**Supplementary Figure 4A**): CDK1 and CCNB1 exhibited focal enrichment in discrete cell clusters, while CDKN1A showed a more widespread but heterogeneous expression profile across multiple cell populations. Quantitative expression analysis across annotated cell types further delineated these patterns (**Supplementary Figure 4B**). To establish a direct link between ZNF695 and G2M checkpoint regulation, we performed correlation analysis between ZNF695 expression and the transcript levels of CDK1, CCNB1, and CDKN1A at the single-cell level (**Supplementary Figure 4C**). We observed strong positive correlations for the positive G2M regulators CDK1 and CCNB1, consistent with ZNF695 driving G2M progression. For the negative regulator CDKN1A, we detected a moderate positive correlation (R = 0.60, p = 2.85e-01) with ZNF695 expression, which may reflect a compensatory feedback mechanism in ZNF695-high cells to restrain unchecked cell cycle progression. Collectively, these single-cell analyses demonstrate the cell-type-specific expression of ZNF695 (enriched in tissue stem cells, epithelial cells, and macrophages) and reveal robust intercellular crosstalk among these ZNF695-high populations in UCEC, suggesting ZNF695 may participate in tumor microenvironment regulation via these cell subsets. These findings indicate that ZNF695 may drive oncogenic pathway activation in UCEC, and its inhibition could attenuate these pro-tumorigenic programs, further supporting the role of ZNF695 as a key regulator of tumor progression and microenvironment remodeling.

### 3.4 ZNF695 regulates key functional pathways in UCEC

**Figure 4:**
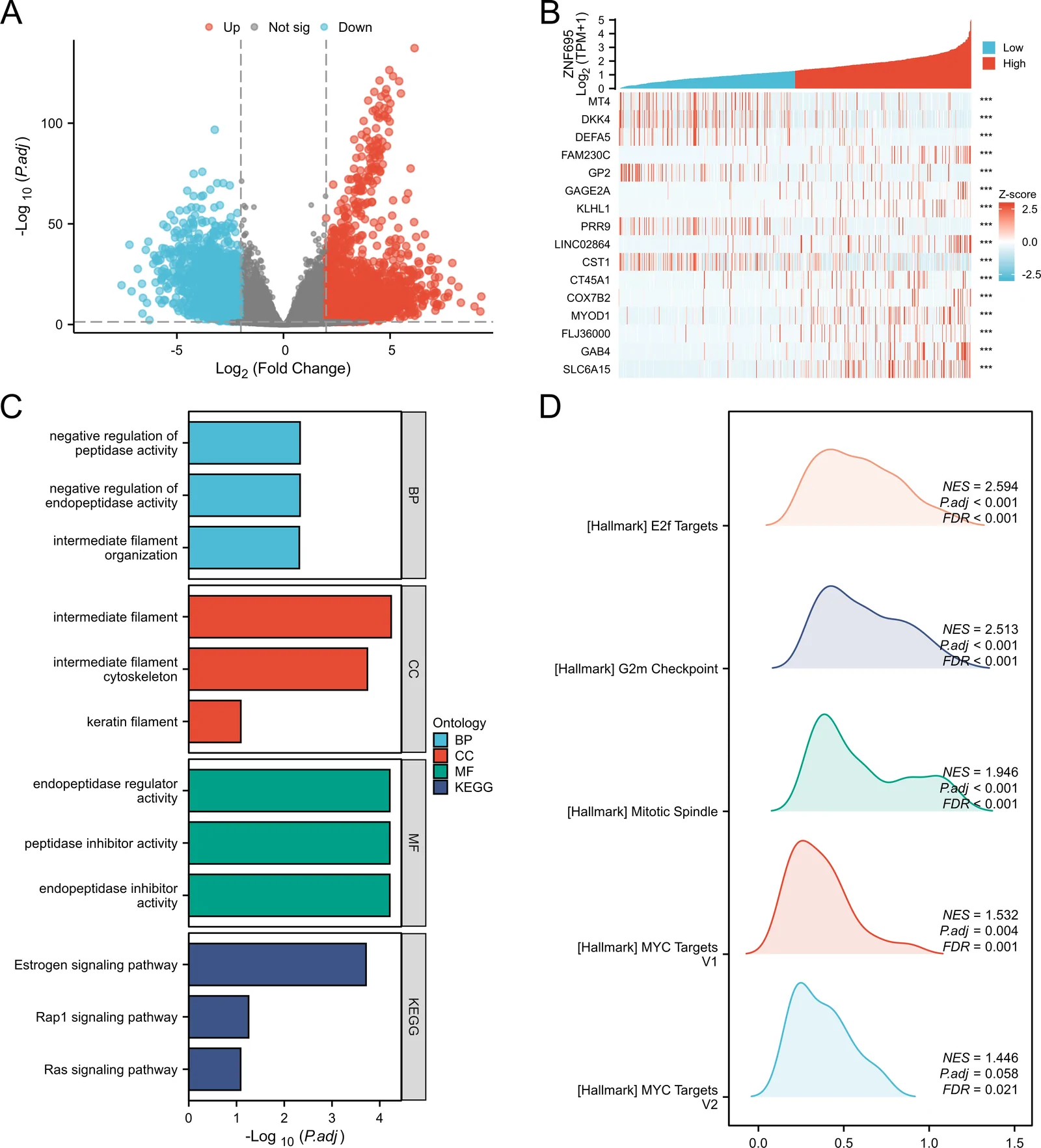
Identification of ZNF695-related DEGs and subsequent analyses. **(A)** Volcano plot presentation of the DEGs. **(B)** Correlations analysis between the top 16 DEGs and ZNF695. **(C)** GO/KEGG analysis. **(D)** GSEA analysis.

A total of 312 genes presented varying levels of expression when comparing ZNF695-high group with ZNF695-low expression group. Among these genes, 181 (58.0%) were upregulated, whereas 131 (42.0%) were downregulated, on the basis of the criteria that Padj was lower than 0.05 and that |Log2-FC| exceeded 2 (**Figure 4A**). The relationships among the top 16 DEGs, including *MT4*, *DKK4*, *DEFA5*, *FAM230C*, *GP2*, *GAGE2A*, *KLHL1*, *PRR9*, *LINC02864*, *CST1*, *CT45A1*, *COX7B2*, *MYOD1*, *FLJ36000*, *GAB4*, and *SLC6A15*, with ZNF695 were analyzed, as presented in **Figure 4B**. Gene ontology and KEGG analyses suggested that these genes were markedly enriched in various essential categories. These included the response mechanisms to the suppression of peptidase activity, intermediate filament, endopeptidase regulator activity, and the estrogen signaling pathway (**Figure 4C**). Our further GSEA analysis indicated ZNF695-related Myc targets V1 and V2, mitotic spindle, G2M checkpoints, and E2F targets (**Figure 4D**). Collectively, these results highlight the unique gene expression signature associated with ZNF695 expression levels, indicating that these DEGs might be integrated with the biological processes associated with UCEC.

### 3.5 A ZNF695-related gene signature predicts prognosis in UCEC

**Figure 5:**
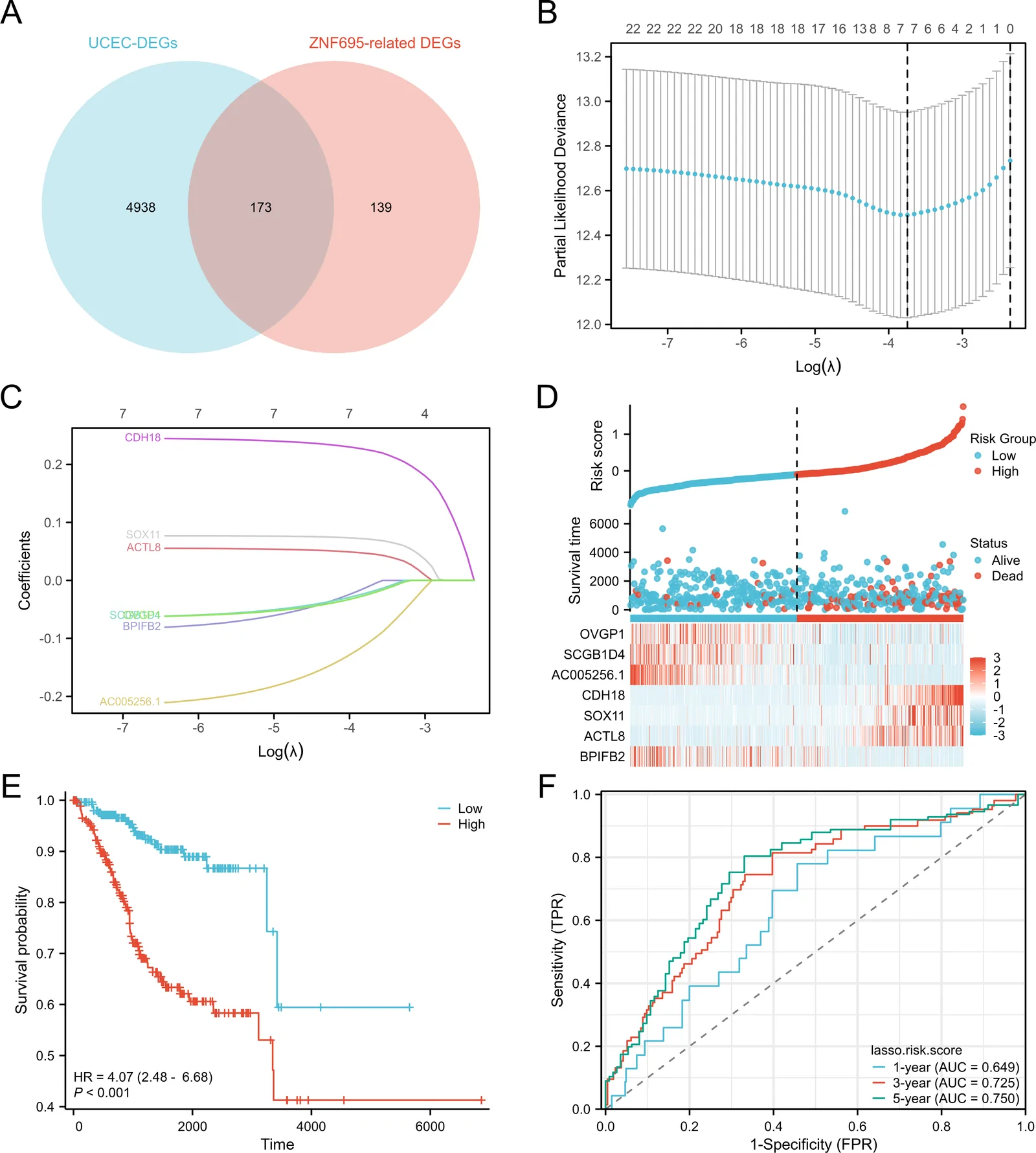
Identification of ZNF695-related prognostic markers in UCEC. **(A)** Venn diagram. **(B)** LASSO risk score. **(C)** Coefficient profiles of 7 candidate genes. **(D)** Expression heatmap of 7 candidate genes. **(E)** Survival analysis. **(F)** ROC curves of the risk model.

To further elucidate the prognostic implications of ZNF695-associated DEGs in UCEC, we identified the overlapping genes between ZNF695-related DEGs and UCEC-related DEGs. A total of 173 overlapping genes were obtained for downstream analytical procedures (**Figure 5A**). We applied LASSO regression to screen for prognostic genes among the overlapping DEGs (**Figure 5B**). A set of key genes (*OVGP1*, *SCGB1D4*, *AC005256.1*, *CDH18*, *SOX11*, *ACTL8*, and *BPIFB2*) that contributed to the prognostic model were identified (**Figure 5C**). The expression profiles of these genes in TCGA-UCEC were presented in **Supplementary Figure 5**. A prognostic risk model was developed to stratify UCEC patients into high- and low-risk cohorts, and we analyzed the distribution of the risk scores, survival status, and the expression heatmap of the model genes (**Figure 5D**). High-risk cohort presented significantly poorer OS result compared with low-risk cohort, as indicated by the Kaplan-Meier survival curve (**Figure 5E**, P < 0.001). Additionally, ROC analysis indicated that our model had excellent predictive performance, with AUC values of 0.649, 0.725, and 0.750 for 1-, 3-, and 5-year OS (**Figure 5F**). Collectively, these results demonstrate that the ZNF695-related gene signature is a reliable prognostic indicator for UCEC patients.

### 3.6 ZNF695 exhibits distinct methylation and mutation profiles in UCEC

**Figure 6:**
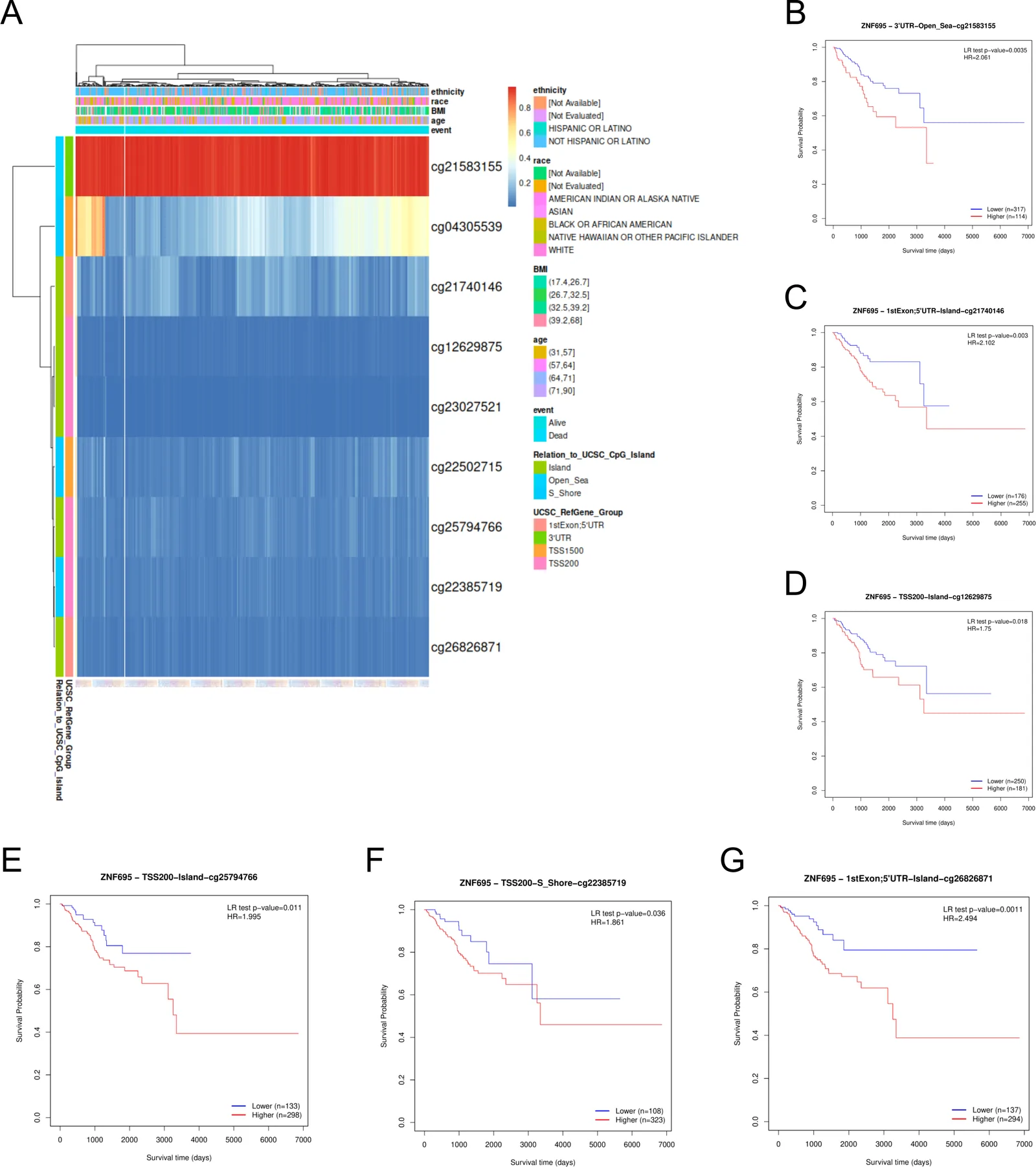
Association between ZNF695 CpG methylation and patient survival. **(A)** Basic annotations of ZNF695-related CpG sites. Survival curves showing the association between methylation levels of ZNF695-specific CpG sites **(B)** cg21583155. **(C)** cg21740146. **(D)** cg12629875. **(E)** cg25794766. **(F)** cg22385719. **(G)** cg26826871.

Methylation patterns of ZNF695 in UCEC were analyzed using the MethSurv database, which identified multiple CpG sites with distinct genomic locations (**Figure 6A**). These sites included cg21583155, cg21740146, cg12629875, cg25794766, cg22385719, and cg26826871. Survival analysis stratified by methylation levels (low vs. high) revealed the prognostic relevance of these ZNF695-related CpG sites (**Figures 6B-G**). Among them, cg12629875, cg25794766, cg22385719, and cg26826871 showed notable associations with OS in UCEC, with differences in survival curves observed between the lower and higher methylation groups over follow-up periods up to 7000 days. These findings suggest that ZNF695 methylation at specific CpG loci may serve as potential prognostic biomarkers for UCEC. Genetic alteration data of ZNF695 in UCEC were retrieved from the cBioPortal database, including datasets from TCGA Firehose Legacy and TCGA PanCancer Atlas (**Supplementary Figure 6**). The mutation spectrum of ZNF695 was dominated by missense mutations of unknown significance, with additional genetic events including amplification and deep deletion. Notably, copy number variation and mutation profiling were available for most samples in the analyzed cohorts. These results indicated that ZNF695 exhibited limited genetic alterations in UCEC, and its oncogenic role may be more closely associated with epigenetic regulation (e.g., methylation) rather than genetic mutations.

### 3.7 ZNF695 expression correlates with immune infiltration in UCEC

**Figure 7:**
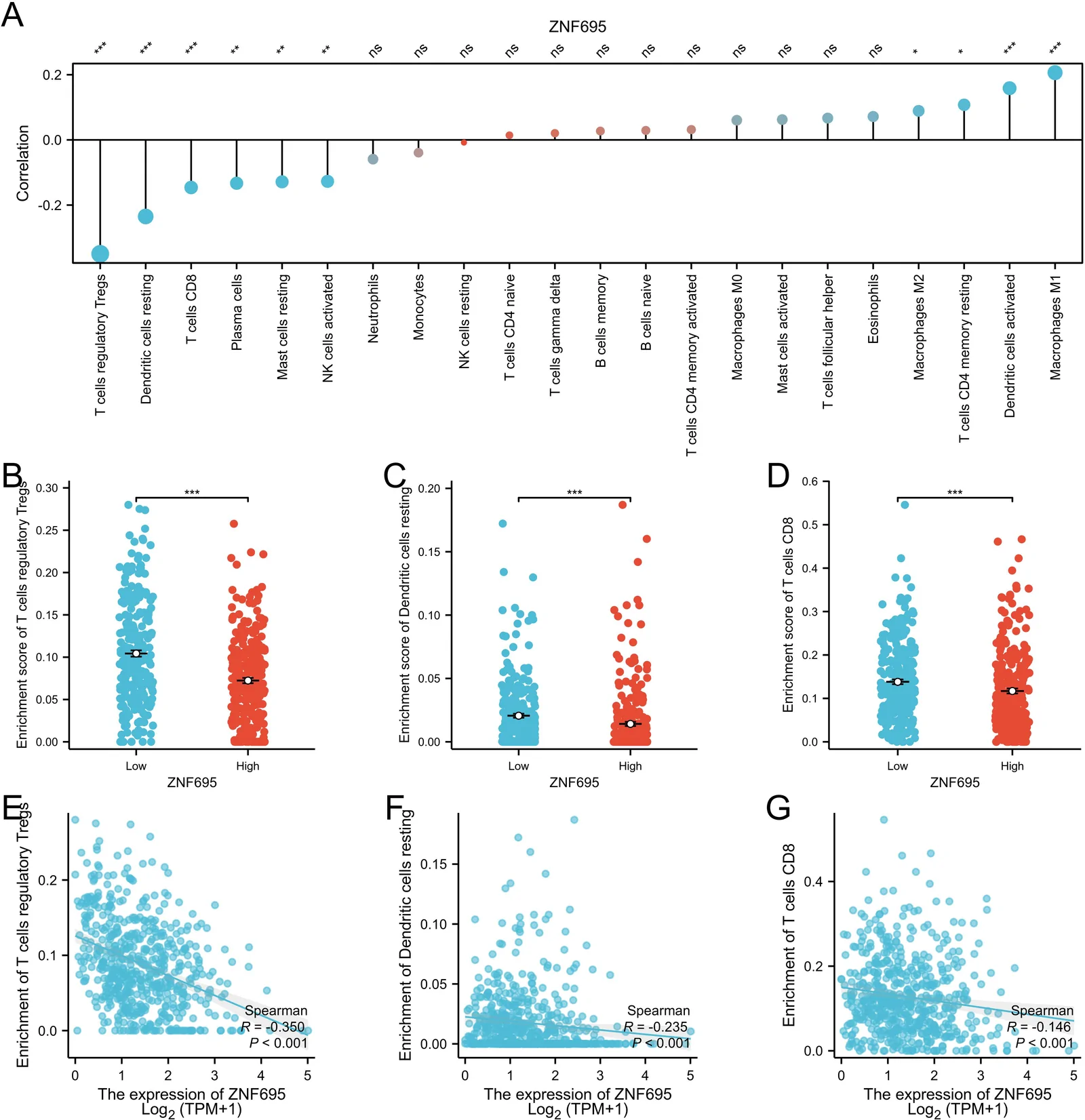
ZNF695 expression correlates with immune infiltration in UCEC. **(A)** Correlation of ZNF695 expression levels with various immune cell types. **(B)** Infiltration differences of T cell regulatory. **(C)** Dendritic cells resting. **(D)** CD8+ T cells. **(E)** Correlation of ZNF695 expression with T cell regulatory. **(F)** Dendritic cells resting. **(G)** CD8+ T cells. * p<0.05, ** p<0.01, *** p<0.001.

To examine the role of ZNF695 expression on the infiltration of diverse immune cell types, we used the CIBERSORT method and examined associations via the Spearman`s rank correlation test (**Figure 7A**). The results yielded a substantial inverse associations between ZNF695 expression and the infiltration of regulatory T cells, resting dendritic cells, and CD8+ T cells (**Figures 7B–G**). Given the critical role of these immune cell populations in antitumor immunity, their reduced abundance indicates that high ZNF695 expression could drive the suppression of immune surveillance pathways in UCEC. On the other hand, a positive association was identified between ZNF695 expression and the infiltration of macrophages M1, dendritic cells activated, and CD4+ T cells resting (**Supplementary Figure 7**). An additional evaluation of immune infiltration was conducted using the ssGSEA method (**Supplementary Figure 8A**). Our findings indicate reverse correlations between ZNF695 and T-cell regulatory Tregs and positive correlations with activated dendritic cells, and monocytes (**Supplementary Figures 8B–D**). We further analyzed the correlations between ZNF695 expression and 11 immune checkpoint genes (**Supplementary Figure 9A**). A weak positive correlation was observed between ZNF695 and SIRPA, while significant negative correlations were found between ZNF695 and PDCD1, CTLA4, and VSIR (**Supplementary Figures 9B–E**). These results demonstrate that ZNF695 might remodulate the tumor microenvironment by inhibiting various immune cell types involved in cytotoxic responses, and promoting the prevalence of immune- tolerant cell types, potentially facilitating tumor immune evasion and progression in UCEC.

### 3.8 High ZNF695 expression is correlated with poor survival in UCEC

**Figure 8:**
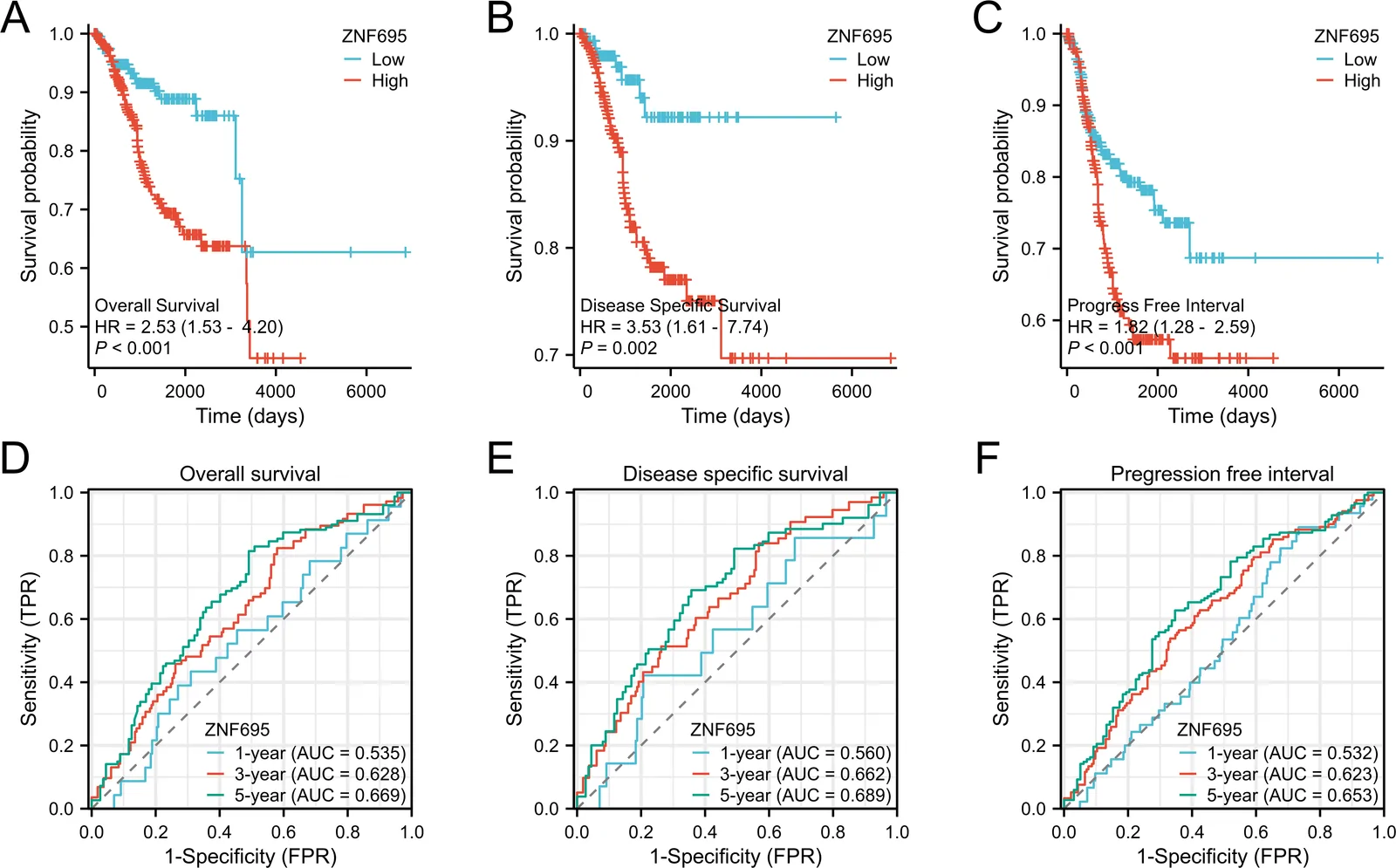
ZNF695 expression is associated with reduced survival in UCEC. **(A)** OS. **(B)** DSS. **(C)** PFI. **(D)** ROC curve for OS. **(E)** ROC curve for DSS. **(F)** ROC curve for PFI.

The survival analysis demonstrated that patients exhibiting elevated expression of ZNF695 experienced poorer OS outcomes (p < 0.001), diminished DSS (p = 0.002), and reduced PFI (p < 0.001) (**Figures 8A–C**). Subgroup analyses UCEC patients also confirmed ZNF695 as an independent prognostic indicator, including age (>60), weight (≤80), BMI (> 30), histologic grade (G1 and G2), clinical stage (stage I and II), histological type (endometrial), menopause status (post), primary therapy outcome (CR and PR), and hormone therapy (post) (**Supplementary Figures 10A–I**). The prognostic relevance of ZNF695 was also investigated across a spectrum of other malignancies with respect to OS, DSS and PFI. The results indicated that ZNF695 also has prognostic value in adrenocortical cancer, cervical cancer, liver cancer, sarcoma, and melanoma (**Supplementary Figures 11A–C**). Our ROC curve analyses showed the AUC score of prognostic value of ZNF695 in 1-, 3-, and 5-year periods (**Supplementary Figures 11D–I****).** These results indicated that ZNF695 may have prognostic value in the context of UCEC and other forms of cancer.

### 3.9 Development and validation of prognostic nomograms in UCEC

**Figure 9:**
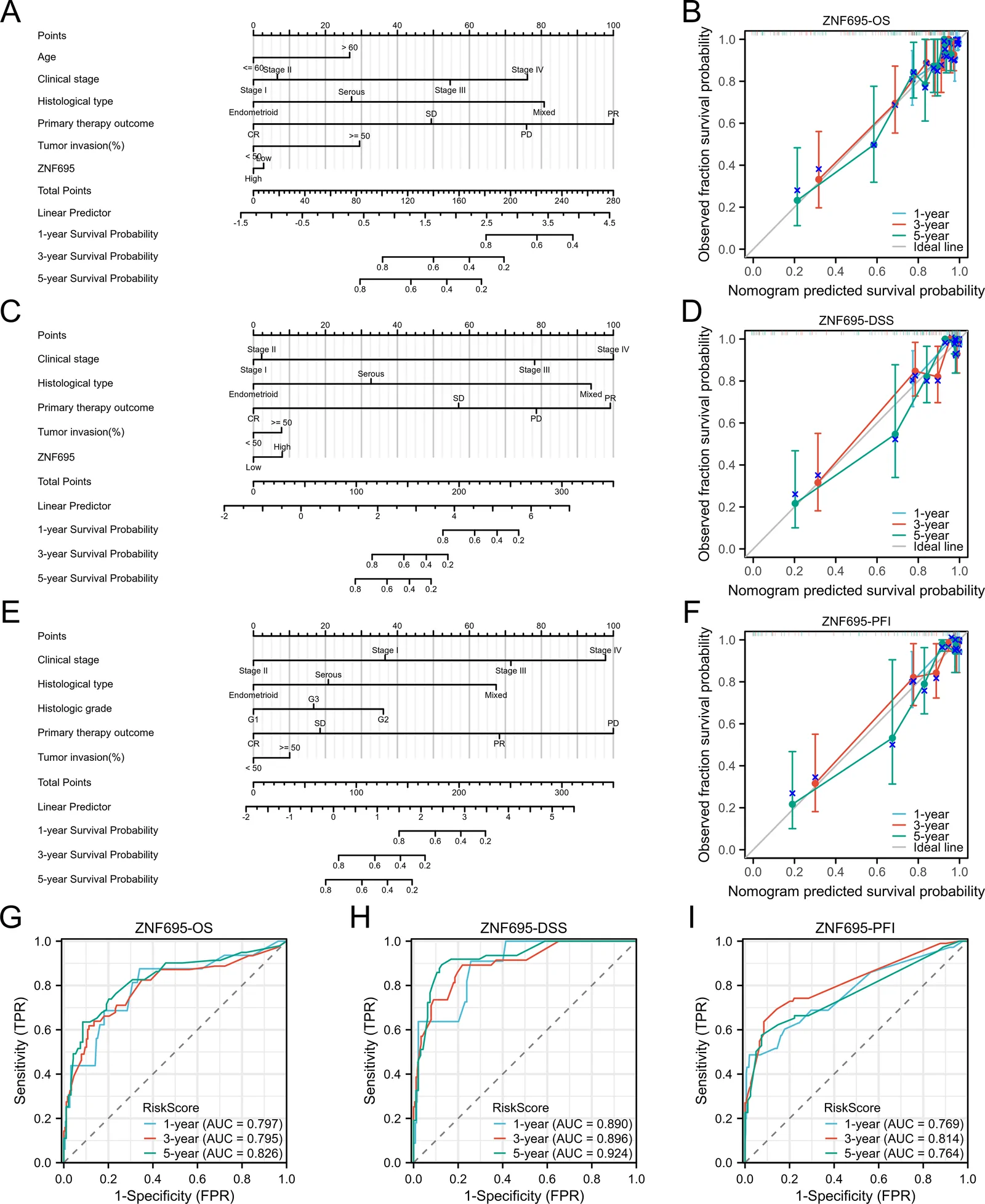
Nomograms and calibration curves of rates of UCEC patients over 1-, 3-, and 5-years. **(A)** Nomogram for 1-, 3-, and 5-year OS in UCEC. **(B)** Calibration curve for the prediction of OS in UCEC. **(C)** Nomogram for 1-, 3-, and 5-year DSS in UCEC. **(D)** Calibration curve for evaluating DSS in UCEC. **(E)** Nomogram for the prediction of 1-, 3-, and 5-year PFI in UCEC. **(F)** Calibration curve for PFIs in UCEC. **(G)** Roc curves fir the OS nomogram. **(H)** Roc curves fir the DSS nomogram. **(I)** Roc curves fir the PFI nomogram.

To offer clinicians with more accurate and personalized prognostic models for UCEC, we constructed 3 nomograms integrating independently validated prognostic variables. These nomograms presented higher cumulative scores to variables correlated with unfavorable survival, therefore providing a visual representation of the risk indications with several clinical and molecular variables. The nomograms were validated via calibration curves, which revealed excellent accuracy of OS rates, (C-index =0.631, **Figures 9A, B**); DSS with a C-index of 0.719 (**Figures 9C, D**); and PFI (C-index=0.787, **Figures 9E, F**). To further assess the predictive performance of the ZNF695-based nomograms, we performed time-dependent ROC curve analyses for OS, DSS, and PFI at 1-, 3-, and 5-year follow-up time points (**Figures 9G–I**). Collectively, these time-dependent ROC curve analyses confirmed that our ZNF695-based nomograms possess excellent predictive accuracy and discriminative ability for OS, DSS, and PFI in UCEC patients, further supporting their clinical utility as reliable prognostic tools. Our univariate Cox analysis revealed that pathologic stage, pathologic T stage, tumor status, and ZNF695 were predictors of OS in patients with UCEC (**Supplementary Table 4**). We also conducted Cox univariate-multivariate analyses for DSS, and PFI (**Supplementary Tables 5**, **6**). These results function as a potential indicator of risk and that the impact of this factor might be subject to modulation by tumor progression and status.

### 3.10 ZNF695 modulates the proliferative capacity of UCEC cells

**Figure 10:**
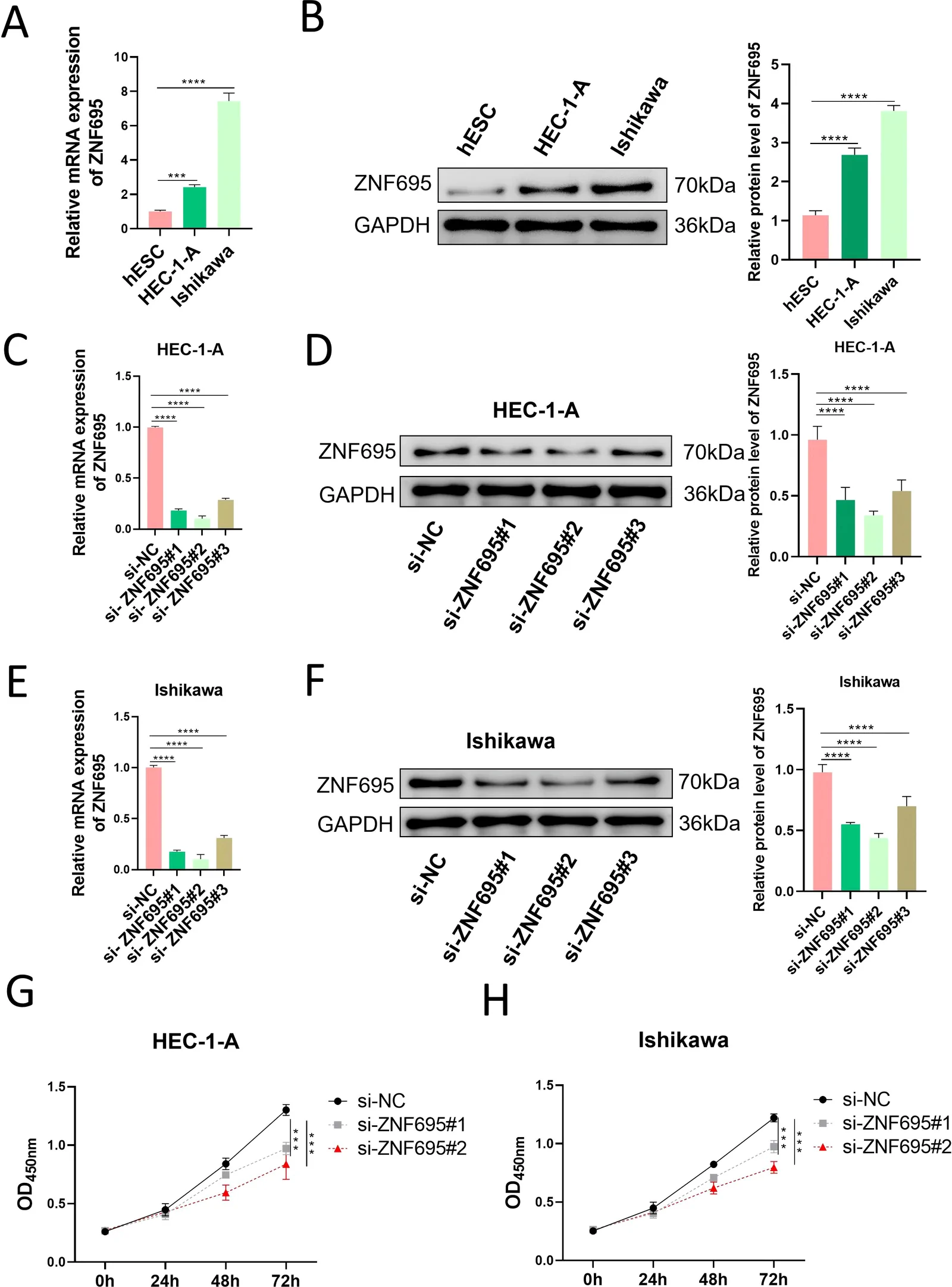
Expression profile of ZNF695 in UCEC cell lines. **(A)** Relative mRNA expression. **(B)** Relative protein expression levels of ZNF695 in UCEC cell lines. **(C)** Knocking down ZNF695 expression in HEC-1-A cells, as measured by qRT-PCR. **(D)** Western blot. **(E)** Knockdown efficiency of ZNF695 siRNAs in Ishikawa cells, assessed by qRT-PCR. **(F)** Western blot. **(G)** Cell proliferation of HEC-1-A. **(H)** Cell proliferation of Ishikawa. ***, P < 0.05, ****, P < 0.0001; ns, not significant (P > 0.05).

To explore the expression pattern of ZNF695 in UCEC cells, we first examined its mRNA and protein levels in the normal human endometrial cell line hESC and two endometrial cancer cell lines (HEC-1-A and Ishikawa) using qRT-PCR and Western blotting. The results showed that both mRNA and protein expression of ZNF695 were significantly higher in HEC-1-A and Ishikawa cells compared with hESC cells (**Figures 10A, B**). To investigate the biological function of ZNF695, three independent small interfering RNAs (siRNAs) targeting ZNF695 (si-ZNF695#1, si-ZNF695#2, and si-ZNF695#3) were transfected into HEC-1-A and Ishikawa cells, with non-targeting siRNA (si-NC) serving as a negative control. qRT-PCR and Western blotting assays confirmed that all three siRNAs effectively knocked down ZNF695 expression at both mRNA and protein levels in HEC-1-A and Ishikawa cells, among which si-ZNF695#1 and si-ZNF695#2 exhibited the highest knockdown efficiency and were thus selected for subsequent functional experiments (**Figures 10C–F**). The effect of ZNF695 silencing on UCEC cell proliferation was then evaluated using CCK-8 assays. The results demonstrated that knockdown of ZNF695 significantly inhibited the proliferation of both HEC-1-A and Ishikawa cells compared with the si-NC group, and this inhibitory effect was time-dependent, becoming more pronounced at 72 h post-transfection (**Figures 10G, H**). Collectively, these findings suggest that ZNF695 is upregulated in UCEC cells and plays a critical role in promoting UCEC cell proliferation *in vitro*.

### 3.11 Silencing ZNF695 suppresses malignant phenotypes in UCEC

**Figure 11:**
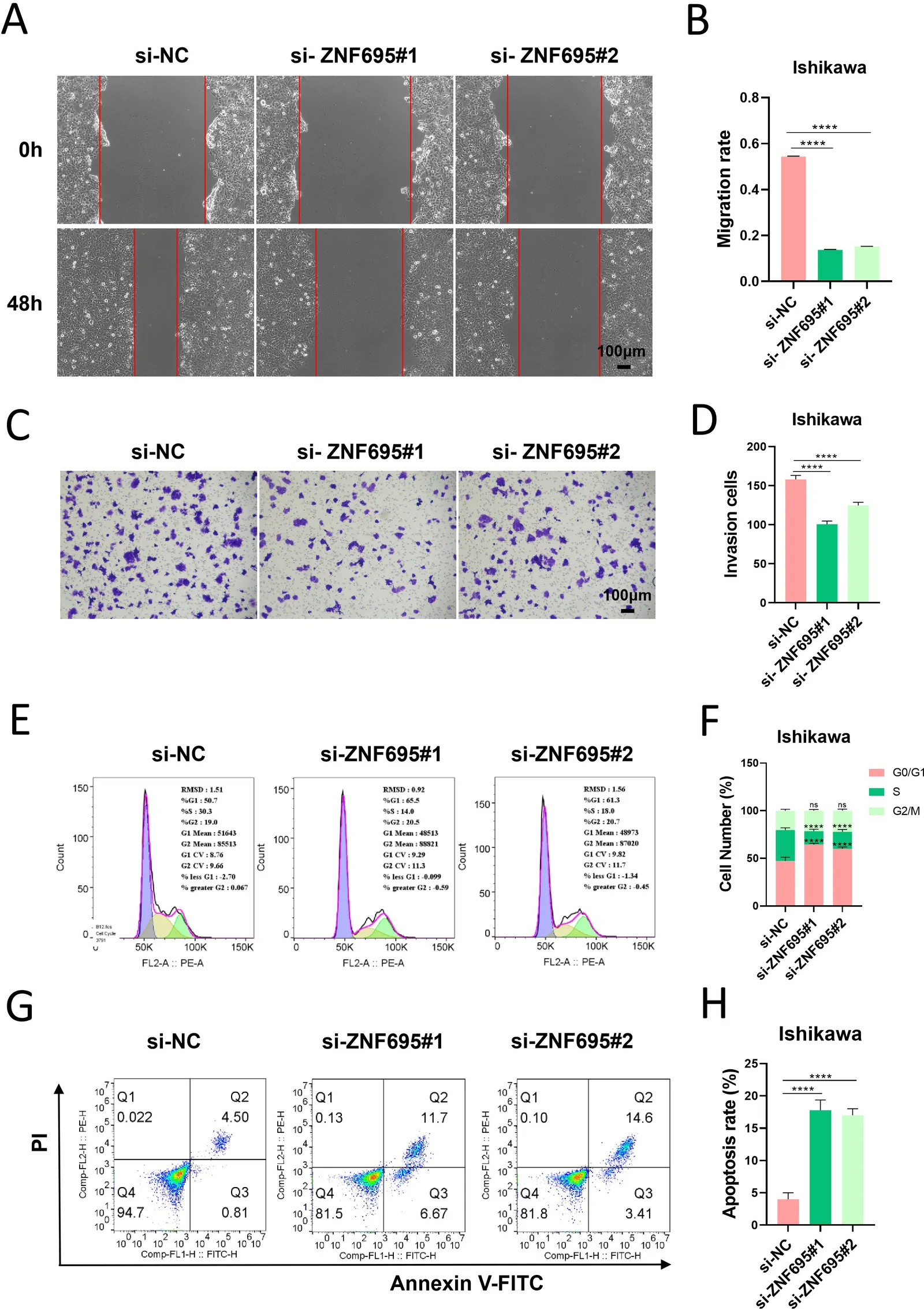
Knockdown of ZNF695 suppresses migration and invasion, induces cell cycle arrest, and promotes apoptosis in Ishikawa cells. (A) Wound-healing assay showing the migratory ability of Ishikawa cells transfected with si-NC, si-ZNF695#1, or si-ZNF695#2. **(B)** Quantitative analysis of migration rates. **(C)** Transwell invasion assay evaluating the invasive capacity of Ishikawa cells after ZNF695 knockdown. **(D)** Quantification of invaded cells. **(E)** Flow cytometric analysis of cell cycle distribution in Ishikawa cells transfected with si-NC, si-ZNF695#1, or si-ZNF695#2. **(F)** The percentage of cells in G0/G1, S, and G2/M phases. **(G)** Representative dot plots. **(H)** The apoptosis rates. ****, P < 0.0001; ns, not significant (P > 0.05).

We further investigated the effect of ZNF695 knockdown on the migration, invasion, cell cycle distribution, and apoptosis of Ishikawa cells. Wound healing assays showed that the migration rate of Ishikawa cells was significantly reduced in both si-ZNF695#1 and si-ZNF695#2 groups compared with the si-NC group after 48 h of culture (**Figures 11A, B**). Consistent with these results, Transwell invasion assays revealed that the number of invasive cells was markedly decreased in ZNF695-silenced Ishikawa cells relative to the control group (**Figures 11C, D**). Flow cytometry analysis of the cell cycle demonstrated that knockdown of ZNF695 induced a significant G0/G1 phase arrest, as evidenced by an increased proportion of cells in the G0/G1 phase and a concomitant decrease in the percentage of cells in the S phase compared with the si-NC group (**Figures 11E, F**). Furthermore, Annexin V-FITC/PI double staining showed that the apoptosis rate was significantly elevated in Ishikawa cells transfected with si-ZNF695#1 or si-ZNF695#2 compared with the si-NC control (**Figures 11G, H**). Collectively, these data indicate that silencing ZNF695 inhibits migration and invasion, induces G0/G1 cell cycle arrest, and promotes apoptosis in endometrial cancer cells.

## 4 Discussion

Uterine corpus endometrial carcinoma is a significant health challenge, ranking among the most common gynecologic cancer with a steadily increasing incidence globally (21). Although early-stage disease is often curable with surgery, patients diagnosed with advanced or recurrent UCEC face limited therapeutic options and a bleak prognosis, with a 5-year OS of 40-50%. Critical challenges in current clinical management include the lack of sensitive early diagnostic biomarkers, and limited efficacy of immune checkpoint inhibitors in molecularly unselected populations. These unresolved issues highlight the urgent need to identify novel molecular drivers that could inform improved risk stratification and therapeutic strategies.

As an important member of the zinc finger protein family, ZNF695 has been documented to play oncogenic roles in several malignancies, including cervical cancer, ovarian cancer, esophageal carcinoma, and childhood B-cell acute lymphoblastic leukemia (22). In colorectal cancer, ZNF695 promotes cancer progression by activating the PI3K/Akt/mTOR signaling pathway. However, its function and clinical significance in UCEC remain unexplored. In this study, bioinformatic analysis of the TCGA-UCEC dataset revealed elevated expression of ZNF695 in UCEC. Consistently, our IHC results using UCEC microarrays also demonstrated significantly higher expression of ZNF695 in UCEC tissues. As a transcription factor, ZNF695 is generally expected to localize to the nucleus, whereas our immunohistochemical results revealed its predominant cytoplasmic localization in UCEC specimens, consistent with data from the Human Protein Atlas (www.proteinatlas.org). Such divergent subcellular distribution may arise from differences in protein activation status and tissue-specific biological contexts. Notably, cytoplasmic retention of inactive ZNF695 may represent a regulatory mechanism restricting its nuclear transcriptional activity in UCEC, thereby altering downstream oncogenic signaling cascades and contributing to UCEC progression.

Beyond its diagnostic value, ZNF695 expression was significantly associated with key clinicopathological features, including advanced age, higher body weight, advanced clinical stage, higher histologic grade, deeper myometrial invasion, and postmenopausal status. The strong diagnostic power of ZNF695 was further supported by ROC analysis, which revealed an AUC score of 0.955, emphasizing its promise as a diagnostic biomarker for UCEC. These results indicate that ZNF695 has potential as diagnostic biomarker in UCEC.

Single-cell RNA sequencing (scRNA-seq) is a useful method that enables the examination of gene expression on a cell-by-cell basis, offering valuable perspectives on the diversity of cellular heterogeneity within complex tissues (23). Our analysis of scRNA-seq data provides novel insights into the cellular context of ZNF695 expression. The prominent expression of ZNF695 in tissue stem cells, epithelial cells and macrophages indicates its vital function within the tumor microenvironment. Macrophages exert dual context-dependent effects on tumor progression: M1-polarized populations typically mediate anti-tumor immunity, while M2-like tumor-associated macrophages facilitate tumor growth and immune evasion (24). The high ZNF695 expression in macrophages raises the intriguing possibility that ZNF695 modulates macrophage polarization or function in UCEC. If ZNF695 indeed drives a pro-tumor phenotypic shift in macrophages, it could contribute to impaired anti-tumor immunity and poor clinical outcomes — a hypothesis supported by the robust intercellular crosstalk between macrophages, epithelial cells, and endothelial cells. Future studies may focus on verifying the polarization state of ZNF695-high macrophages in UCEC and dissecting the molecular pathways through which ZNF695 regulates macrophage functions.

Our functional pathway analysis revealed that ZNF695 was associated with several biological processes and oncogenic signaling pathways, notably estrogen signaling and cell cycle regulation. Differentially expressed genes (DEGs) correlated with ZNF695 showed significant enrichment in functions pertinent to peptidase activity and cell cycle control, suggesting a potential influence of ZNF695 on tumor growth and metastatic behavior. This was corroborated by our GSEA results, which demonstrated significant enrichment of gene sets related to critical cell cycle checkpoints and DNA replication. These results imply that ZNF695 may contribute to UCEC pathogenesis by modulating cell proliferation and survival mechanisms.

Immune cell infiltration is a pivotal determinant in cancer progression and governing the functional balance of immune response within the tumor microenvironment (25). In the current study, our exploration of ZNF695-related immune infiltration revealed complex associations between ZNF695 expression and the infiltration of several immune cells. The negative associations between ZNF695 levels and the infiltration of regulatory T cells and CD8+ T cells suggests that high ZNF695 expression may contribute to an immunosuppressive tumor microenvironment. This finding is particularly relevant as Tregs and CD8+ T cells are critical for antitumor immunity. Conversely, the positive correlation with M1 macrophages and activated dendritic cells indicates that ZNF695 may also promote certain immune responses that could be harnessed for therapeutic benefit. The balance between these opposing effects underscores the need for further research to clarify the specific function of ZNF695 in immune modulation within UCEC.

Our survival analysis provides compelling evidence for the prognostic significance of ZNF695 in UCEC. The observation that high ZNF695 expression correlates with worse OS, DSS, and PFI underscores its potential as a prognostic biomarker. This was substantiated by Cox regression analysis, which identified ZNF695 as an independent prognostic factor, suggesting that it could be integrated into clinical practice to enhance clinical risk stratification of UCEC patients. The development of prognostic nomograms incorporating ZNF695 and other clinical parameters offers a valuable tool for personalized management of patients, enabling clinicians to make informed treatment decisions based on individual risk profiles.

The limitations of the present study its dependence on data from the TCGA-UCEC cohort and the absence of *in vivo* validation of ZNF695 in UCEC. Additionally, the analysis was based on a relatively small sample size, and scRNA-seq data were derived from only one GEO dataset with only 5 patient samples. Further research should expand the sample size by integrating multi-center clinical datasets to improve the reliability of the findings. Moreover, comprehensive cell and animal experiments combined with mechanistic validations are necessary to clarify the functional role and molecular functions of ZNF695 in UCEC.

## 5 Conclusion

In summary, our research establishes ZNF695 as a critical regulatory factor in UCEC, influencing tumor biology, immune interactions, and patient outcomes. The high expression of ZNF695 in UCEC and its correlation with key clinical characteristics and prognostic stratification underscore its potential as a promising biomarker. Subsequent research should aim to examine the molecular functions underlying ZNF695’s effects on tumor development, metastasis, and immune evasion, as well as explore its potential in combination therapies aimed at enhancing antitumor immunity.

## Data Availability

The original contributions presented in the study are included in the article/**Supplementary Material**. Further inquiries can be directed to the corresponding authors.
