# Supplementary material for: Prognostic significance of ZNF695 in uterine corpus endometrial carcinoma: single-cell and immune infiltration analyses
Source: Front Oncol. 2026 Aug 10;16:1827186. doi: 10.3389/fonc.2026.1827186 (PMC13501525; doi:10.3389/fonc.2026.1827186)
Supplement: Supplementary file 1 [file DataSheet1.docx]

**Supplementary materials**

Supplementary Figure 1

Supplementary Figure 1. Pan cancer analysis of ZNF695. (A) TIMER 2.0 database. (B) TCGA data. (C) TCGA data (paired).

Supplementary Figure 2

Supplementary Figure 2. ZNF695 expression in GEO database. (A) GSE183185. (B) GSE106191. (C) ROC value of ZNF695 in GSE183185. (D) ROC value of ZNF695 in GSE106191.

Supplementary Figure 3

Supplementary Figure 3. The expression of ZNF695 in UCEC tissue microarray. (A) UCEC tissue microarray. (B) ZNF695 expression in UCEC (10x). (C) ZNF695 expression in UCEC (40x). (D) ZNF695 expression in adjacent normal tissues (10x). (E) ZNF695 expression in adjacent normal tissues(40x). (F)CDKN2A. (G) PGR. (H) MKI67.

Supplementary Figure 4

Supplementary Figure 4. Single-cell expression profiles and correlation analysis of CDK1, CCNB1, and CDKN1A with ZNF695. (A) UMAP plots showing single-cell expression distribution of CDK1, CCNB1, and CDKN1A across all cell populations. (B) Violin plots illustrating CDK1, CCNB1, and CDKN1A expression levels in distinct UCEC cell subsets. (C) Scatter plots presenting the correlation between ZNF695 and CDK1, CCNB1, and CDKN1A expression.

Supplementary figure 5

Supplementary Figure 5. Expression of ZNF695-related prognostic genes in TCGA-UCEC cohort. (A) CDH18. (B) SOX11. (C) ACTL8. (D) BPIFB2. (E) AC005256.1. (F) OVGP1.

Supplementary figure 6

Supplementary Figure 6. Mutation status of ZNF695 in UCEC. (A) Clinical/genomic feature summary (mutation, CNV, prognosis) of ZNF695 in UCEC. (B) Genomic alteration landscape of ZNF695 in TCGA-UCEC cohort. (C) Overall survival. (D) Disease free survival. (E) Disease specific survival. (F) Progression free survival.

Supplementary figure 7

Supplementary Figure 7. The correlation of ZNF695 with various immune cells. (A) Macrophages M1. (B) Activated dendritic cells. (C) T cells CD4 memory resting.

Supplementary Figure 8

Supplementary Figure 8. ssGSEA analysis of ZNF695 expression and various immune cells. (A) The correlations between ZNF695 expression and 23 types of immune cells. (B) NK CD56bright cells. (C) iDC. (D) pDC. (E) Th2 cells. (F) T helper cells. (G) Tcm.

Supplementary Figure 9

Supplementary Figure 9. The correlation of ZNF695 with 11 immune checkpoint genes. (A) Heatmap overview. (B) SIRPA. (C) PDCD1. (D) CTLA4. (E) VSIR.

Supplementary Figure 10

Supplementary Figure 10. Prognostic significance of ZNF695 in various subgroups in UCEC. (A) Age ≤ 60 years. (B) Weight ≤80. (C) BMI > 30. (D) Clinical stage, I and II. (E) Histologic grade, G1 and G2. (F) Histological type: Endometrical. (G) Primary therapy outcome: CR and PR. (H) Menopause status: Post. (I) Hormone therapy: No.

Supplementary Figure 11

Supplementary Figure 11. Prognostic significance of ZNF695 expression across various cancer types. (A) OS. (B) DSS. (C) PFI. (D) ROC in ACC. (E) ROC in CESC. (F) ROC in LIHC. (G) ROC IN SARC. (H) ROC in SKCM. (I) ROC in UCEC.

**Supplementary Table 1. Sequences of si- ZNF695**

| Gene | SS Sequence | AS Sequence |
| --- | --- | --- |
| si- ZNF695 #1 | 5'- AUUGCAUAUUGAAGCUAUCCU -3' | 5'- GAUAGCUUCAAUAUGCAAUUC -3' |
| si- ZNF695 #2 | 5'- AGGAAUUGCAUAUUGAAGCUA -3' | 5'- GCUUCAAUAUGCAAUUCCUAU -3' |
| si- ZNF695 #3 | 5'- UAGGAAUUGCAUAUUGAAGCU -3' | 5'- CUUCAAUAUGCAAUUCCUAUU -3' |
| si-NC | 5'- UUCUCCGAACGUGUCACGUTT -3 | 5'- ACGUGACACGUUCGGAGAATT -3' |

**Supplementary Table 2. Sequences of primers**

| Gene | Forward sequence | Reverse sequence |
| --- | --- | --- |
| ZNF695 | 5'- TGGAAAGGGCAGAAGGCAAG -3' | 5'- GACATGCAAGAGACATTGCCA -3' |
| GAPDH | 5'- AAAGCCTGCCGGTGACTAAC -3' | 5'- GCCCAATACGACCAAATCAGA -3' |

**Supplementary Table 3. Clinical features of UCEC patients in tissue microarray**

| Patient Number | Age | Diagnosis | Lymph node metastasis (Left) | Lymph node metastasis (Right) | Pelvic Lymph node metastasis | Distant metastasis | TNM | P53 | Ki-67 |
| --- | --- | --- | --- | --- | --- | --- | --- | --- | --- |
| A01 | 62 | UCEC | 0--16 | 0--12 | 0 | no | T1bN0M0 | 1 | 0.1 |
| A03 | 53 | UCEC | 0--20 | 0--22 | 0 | no | T1aN0M0 | 1 | 0.4 |
| A05 | 60 | UCEC | 0--3 | 0--9 | 0 | no | T1bN0M0 | 1 | 0.3 |
| A07 | 47 | UCEC | 0--11 | 0--13 | 0 | no | T1bN0M0 | 1 | 0.5 |
| A09 | 57 | UCEC | 0--3 | 0--10 | 0 | no | T1aN0M0 | 1 | 0.1 |
| A11 | 49 | UCEC | 0--8 | 0--9 | 0 | no | T1bN0M0 | 1 | 0.1 |
| B01 | 56 | UCEC | 0--3 | 0--3 | 0 | no | T1aN0M0 | 1 | 0.6 |
| B03 | 55 | ChCC | 0 | 0 | 0 | no | T1bNxM0 | 1 | 0.8 |
| B05 | 71 | UCEC | 0--7 | 0--10 | 0 | no | T1aN0M0 | 0 | 0.2 |
| B07 | 48 | UCEC | 0 | 0--9 | 0 | no | T1aN0M0 | 0 | 0.5 |
| B09 | 44 | UCEC | 0--8 | 0--6 | 0 | no | T1aN0M0 | 1 | 0.05 |
| B11 | 52 | UCEC | 2--7 | 0--3 | 1 | Rectum | T4N1M1 | 0 | 0.3 |
| C01 | 54 | UHGSC | 0 | 0 | 0 | no | T1aNxM0 | 3 | 0.5 |
| C03 | 67 | UCEC | 0--12 | 0--15 | 0 | no | T1aN0M0 | 1 | 0.4 |
| C05 | 62 | UCEC | 1--10 | 2--11 | 1 | no | T1bN1M0 | 1 | 0.3 |
| C07 | 80 | UCEC | 0 | 0 | 0 | no | T1bNxM0 | 0 | 0.7 |
| C09 | 66 | UCEC | 0--8 | 0--17 | 0 | no | T1aN0M0 | 1 | 0.3 |
| C11 | 49 | UCEC | 0--15 | 0--9 | 0 | no | T1aN0M0 | 1 | 0.5 |
| D01 | 53 | UCEC | 0--27 | 0--36 | 0 | no | T1bN0M0 | 3 | 0.6 |
| D03 | 67 | UCEC | 0--15 | 0--22 | 0 | no | T1aN0M0 | 0 | 0.45 |
| D05 | 49 | UHGSC | 0 | 0--9 | 0 | no | T1bN0M0 | 2 | 0.6 |
| D07 | 69 | UCEC | 0--15 | 0--25 | 0 | no | T1aN0M0 | 1 | 0.6 |
| D09 | 35 | UCEC | 0--12 | 0--16 | 0 | no | T1bN0M0 | 1 | 0.3 |
| D11 | 62 | UCEC | 0--29 | 0--28 | 0 | no | T1aN0M0 | 0 | 0.3 |
| E01 | 64 | UCEC | 0--17 | 0--28 | 0 | no | T1bN0M0 | 1 | 0.5 |
| E03 | 51 | UCEC | 0--8 | 0--9 | 0 | no | T1aN0M0 | 1 | 0.8 |
| E05 | 61 | UCEC | 0 | 0 | 0 | no | T1bNxM0 | 1 | 0.3 |
| E07 | 60 | UCEC | 1--9 | 3--8 | 1 | no | T1bN2M0 | 1 | 0.3 |
| E09 | 39 | UCEC | 0--15 | 0--18 | 0 | no | T1aN0M0 | 0 | 0.2 |
| E11 | 40 | UCEC | 0--18 | 0--24 | 0 | no | T1bN0M0 | 0 | 0.6 |
| F01 | 57 | UCEC | 0--16 | 0--24 | 0 | no | T1bN0M0 | 1 | 0.7 |
| F03 | 67 | UCEC | 0--17 | 0--12 | 0 | no | T1bN0M0 | 1 | 0.7 |
| F05 | 37 | UCEC | 8--10 | 3--3 | 1 | Sigmoid colon | T4N2M1 | 0 | 0.4 |
| F07 | 50 | UCEC | 0--1 | 0--20 | 0 | no | T1aN0M0 | 0 | 0.15 |
| F09 | 73 | UCEC | 0--10 | 0--11 | 0 | no | T1bN0M0 | 1 | 0.1 |
| F11 | 67 | UCEC | 0--18 | 0--20 | 0 | no | T1aN0M0 | 3 | 0.8 |
| G01 | 57 | UCEC | 0--7 | 0--9 | 0 | no | T1bN0M0 | 0 | 0.4 |
| G03 | 49 | UCEC | 0--22 | 0--25 | 0 | no | T1aN0M0 | 0 | 0.4 |
| G05 | 63 | UHGSC | 0--8 | 0--18 | 0 | no | T1aN0M0 | 1 | 0.4 |
| G07 | 46 | MCS | 0--6 | 0--11 | 0 | no | T1aN0M0 | 0 | 0.7 |
| G09 | 61 | UCEC | 0--5 | 0--6 | 0 | no | T1aN0M0 | 1 | 0.1 |
| G11 | 58 | UCEC | 6--11 | 5--19 | 1 | no | T3N2M0 | 1 | 0.5 |
| H01 | 60 | UCEC | 3--3 | 0 | 0 | no | T1bN0M0 | 0 | 0.8 |
| H03 | 27 | UCEC | 0--10 | 0--17 | 0 | no | T1aN0M0 | 1 | 0.25 |
| H05 | 64 | UCEC | 0--4 | 0--5 | 0 | no | T1aN0M0 | 0 | 0.1 |
| H07 | 55 | UCEC | 0--10 | 0 | 0 | no | T1aN0M0 | 3 | 0.6 |
| H09 | 54 | UCEC | 5--20 | 3--23 | 1 | Liver | T1bN2M1 | 1 | 0.6 |
| H11 | 70 | UCEC | 0--17 | 0--17 | 0 | no | T1bN0M0 | 1 | 0.2 |

Abbreviations: Uterine corpus endometrial carcinoma - UCEC; Choriocarcinoma - ChCC;

Uterine high-grade serous carcinoma - UHGSC; Mullerian carcinosarcoma - MCS

**Supplementary Table 4.**

| Characteristics | Total (N) | Univariate | | Multivariate | |
| --- | --- | --- | --- | --- | --- |
|  |  | HR (95% CI) | p | HR (95% CI) | p |
| Age | 551 | 1.850 (1.162 - 2.944) | **0.009** | 1.652 (0.883 - 3.090) | 0.116 |
| Clinical stage | 553 | 8.082 (4.497 - 14.524) | **< 0.001** | 4.609 (1.895 - 11.208) | **< 0.001** |
| Weight | 529 | 1.055 (0.696 - 1.600) | 0.800 |  |  |
| BMI | 520 | 0.963 (0.634 - 1.464) | 0.861 |  |  |
| Histologic grade | 542 | 13.303 (3.262 - 54.242) | **< 0.001** | 3.419 (0.750 - 15.580) | 0.112 |
| Histological type | 553 | 2.674 (1.744 - 4.100) | **< 0.001** | 1.543 (0.788 - 3.021) | 0.206 |
| Tumor invasion | 475 | 2.825 (1.752 - 4.554) | **< 0.001** | 1.837 (0.996 - 3.387) | 0.051 |
| Primary therapy outcome | 482 | 0.110 (0.060 - 0.204) | **< 0.001** | 0.224 (0.098 - 0.509) | **< 0.001** |
| Menopausal status | 506 | 0.842 (0.388 - 1.826) | 0.664 |  |  |
| ZNF695 | 553 | 1.774 (1.167 - 2.696) | **0.007** | 0.940 (0.519 - 1.703) | 0.838 |

**Supplementary Table 5.**

| Characteristics | Total (N) | Univariate | | Multivariate | |
| --- | --- | --- | --- | --- | --- |
|  |  | HR (95% CI) | p | HR (95% CI) | p |
| Weight | 527 | 0.908 (0.548 - 1.503) | 0.708 |  |  |
| BMI | 518 | 0.945 (0.567 - 1.574) | 0.827 |  |  |
| Clinical stage | 551 | 16.848 (8.290 - 34.238) | **< 0.001** | 11.088 (3.595 - 34.200) | **< 0.001** |
| Histological type | 551 | 3.503 (2.077 - 5.909) | **< 0.001** | 1.891 (0.869 - 4.115) | 0.108 |
| Primary therapy outcome | 482 | 0.062 (0.032 - 0.120) | **< 0.001** | 0.127 (0.052 - 0.312) | **< 0.001** |
| Tumor invasion | 475 | 3.296 (1.808 - 6.011) | **< 0.001** | 1.275 (0.581 - 2.799) | 0.545 |
| Menopausal status | 504 | 0.857 (0.343 - 2.139) | 0.740 |  |  |
| Hormones therapy | 346 | 0.789 (0.308 - 2.018) | 0.621 |  |  |
| ZNF695 | 551 | 2.126 (1.259 - 3.591) | **0.005** | 1.178 (0.524 - 2.652) | 0.692 |

**Supplementary Table 6.**

| Characteristics | Total (N) | Univariate | | Multivariate | |
| --- | --- | --- | --- | --- | --- |
|  |  | HR(95% CI) | p | HR(95% CI) | p |
| Weight | 529 | 1.028 (0.723 - 1.463) | 0.877 |  |  |
| BMI | 520 | 1.040 (0.725 - 1.491) | 0.831 |  |  |
| Clinical stage | 553 | 6.852 (4.093 - 11.471) | **< 0.001** | 3.944 (1.913 - 8.130) | **< 0.001** |
| Histological type | 553 | 2.132 (1.471 - 3.091) | **< 0.001** | 1.547 (0.894 - 2.677) | 0.119 |
| Histologic grade | 542 | 3.304 (1.720 - 6.345) | **< 0.001** | 1.281 (0.543 - 3.025) | 0.572 |
| Primary therapy outcome | 482 | 0.059 (0.035 - 0.098) | **< 0.001** | 0.097 (0.049 - 0.194) | **< 0.001** |
| Tumor invasion | 475 | 1.897 (1.298 - 2.773) | **< 0.001** | 1.297 (0.815 - 2.065) | 0.273 |
| Menopausal status | 506 | 1.551 (0.682 - 3.525) | 0.295 |  |  |
| Hormones therapy | 346 | 1.258 (0.704 - 2.245) | 0.438 |  |  |
| ZNF695 | 553 | 1.743 (1.221 - 2.489) | **0.002** | 1.399 (0.852 - 2.299) | 0.185 |
